# High incidence and mortality of COVID-19 among patients with haematological malignancies: an observational study in santander, Colombia

**DOI:** 10.1016/j.nmni.2022.100988

**Published:** 2022-05-27

**Authors:** Yeimer Ortiz-Martínez, Javier E. Fajardo-Rivero, Tania Mendoza-Herrera, Claudia Figueroa-Pineda, Carlos Ruiz-González, Zane Saul, Alfonso J. Rodríguez-Morales

**Affiliations:** 1 Department of Internal Medicine, Universidad Industrial de Santander, Bucaramanga, Santander, Colombia; 2 Department of Infectious Diseases, Yale New Haven Health - Bridgeport Hospital, Bridgeport, CT, USA; 3 Grupo de Investigación Biomedicina, Faculty of Medicine, Fundación Universitaria Autónoma de Las Américas, Pereira, Colombia; 4 Facultad de Ciencias de La Salud, Universidad Científica Del Sur, Lima, Peru

The incidence and prognosis of COVID-19 in patients with haematological malignancies (HM) are of utmost interest due to their high degree of humoral and cellular dysfunction. Previous research suggests that patients with HM experience significant morbidity and mortality resulting from COVID-19 infection compared to the general population [1,2]. A recent study on children with HM suggest they are at no greater risk of severe SARS-CoV-2 infection than those with non-HM [3]. However, there is a limited number of studies about COVID-19 in patients with HM, particularly in Latin America. Therefore, this study aims to report the real-world incidence and outcomes of COVID-19 in patients with HM compared to the general population in Santander, Colombia (Fig. 1).

**Fig. 1:**
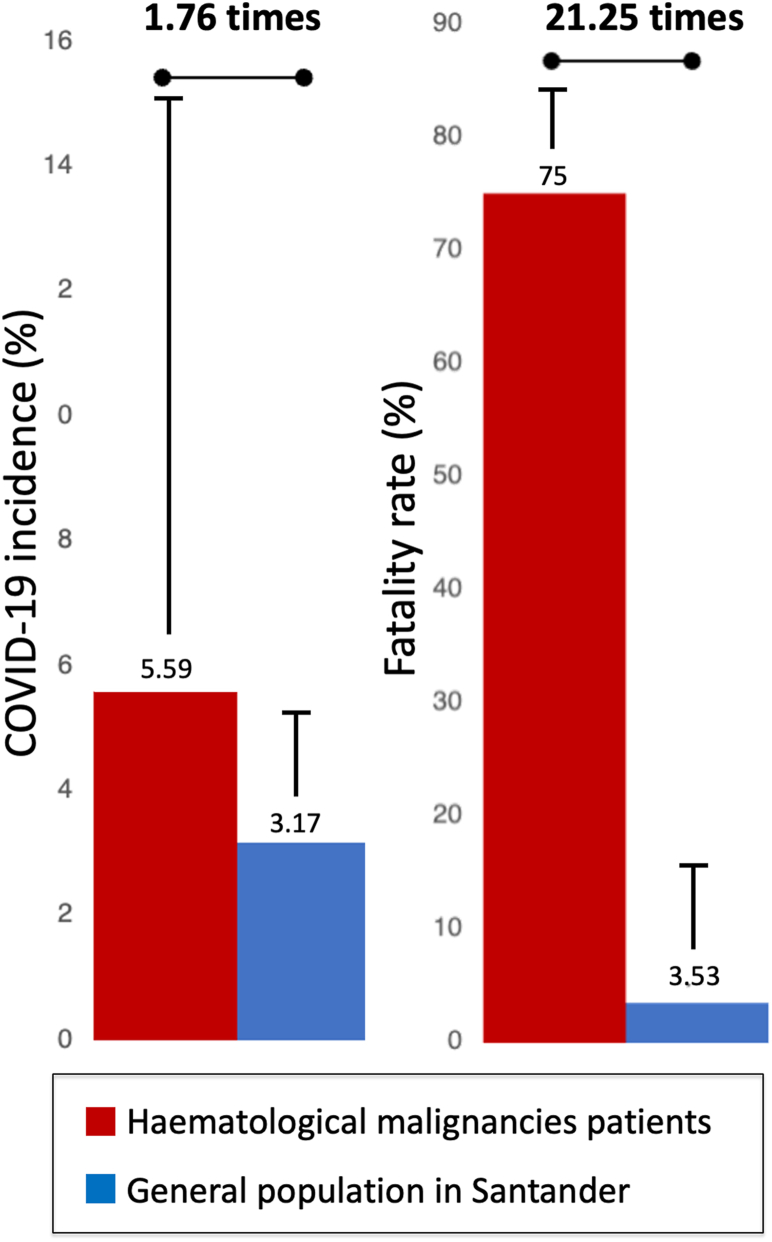
Comparison of COVID-19 incidence and fatality rate among patients with haematological malignancies and the general population of Santander, Colombia, 2020.

An observational, retrospective and cross-sectional study was conducted, which included all hospitalized patients >18 years with a confirmed diagnosis of HM in a reference public tertiary hospital in Bucaramanga, Santander, Northeast Colombia, where SARS-CoV-2 screening is performed on all HM patients, between January and December 2020. We focused on RT-PCR SARS-CoV-2-positive patients, reporting on the clinical characteristics of the HM and the infection. In addition, COVID-19 incidence data of the Santander population was collected through the Epidemiological Surveillance System (SIVIGILA). The protocol was evaluated and approved by Institutional Ethical Committees of the Universidad Industrial de Santander (Act No. 20, meeting on 27 November 2020) and Hospital Universitario de Santander (Act No. 5, meeting on 5 April 2021). Collected data were compiled in Excel and then analyzed with Stata v. 14.0® College Station, TX: StataCorp LP.

We documented eight COVID-19 cases among 143 HM patients, resulting in an accumulated incidence in 2020 of 5.59% (IC95% 1.48-9.7), which was higher than that of the general population in Santander (3.17%) (ratio 1.76). Four were male; the median age was 65.2 years (range 31-79). Three cases were in patients with chronic lymphocytic leukaemia, two with non-Hodgkin's lymphoma, two with multiple myeloma and one with myelodysplastic syndrome. 62.5% had relapsed, or refractory disease and only one patient received chemotherapy during hospitalization. The median in-hospital stay was 17.2 days (range 5-31). There were no differences in the COVID-19 incidence between HM subtypes (*p* = 0.2). The COVID-19-related fatality rate was 75% (6/8), considerably higher than the reported in the general population of Santander (3.53%) (ratio 21.25).

Despite the limitations of our study, including the retrospective nature of the study, the limited number of cases, the lack of power to support firm conclusions and the heterogeneity of HM included; to our knowledge, this is study is among the first exploring the COVID-19 incidence among HM patients compared to the general population in Latin America. There are multiple concerns about in patients with HM regarding COVID-19, as well as with other infectious diseases [4], including with this pandemic threat the low rate of seroconversion in HM patients, that some recent studies suggest that treatment-mediated immune dysfunction is a main driver [5]. Our study suggests an increased risk of SARS-CoV-2 infection and COVID-19-related lethality in patients with haematological malignancies, even without exposure to bone marrow suppressing drugs, compared to the general population, especially those with relapsed disease. It supports the high vulnerability of those patients in the current pandemic and the need for increased surveillance, universal SARS-CoV-2 immunization, screening and possible protective isolation.

## Disclosure statement

No potential conflict of interest was reported by the author(s).

## Funding source

None.

## Author contributions

YOM: Conceptualisation, Methodology, Writing – Original draft preparation; JEFV, TMH, CFL, CRG, ZS, AJRM: Data curation, Writing – Reviewing and editing. All authors read and approved the final manuscript.
